# Surveillance, Self and Smartphones: Tracking Practices in the Nightlife

**DOI:** 10.1007/s11948-015-9691-8

**Published:** 2015-08-30

**Authors:** Tjerk Timan, Anders Albrechtslund

**Affiliations:** 1 Tilburg University, Tilburg, The Netherlands; 2 Aarhus University, Aarhus, Denmark

**Keywords:** Surveillance, Tracking, Self, Smartphones, Nightlife

## Abstract

This paper is the result of the EMERGING ICT FOR CITIZEN VEILLANCE-workshop organized by the JRC, Ispra, Italy, March 2014. The aim of this paper is to explore how the subject participates in surveillance situations with a particular focus on how users experience everyday tracking technologies and practices. Its theoretical points of departure stem from Surveillance Studies in general and notions of participatory surveillance (Albrechtslund 2008) and empowering exhibitionism (Koskela in Surveill Soc 2(2/3):199–215, 2004) in particular. We apply these theoretical notions on smartphones and its users to investigate the combination of participation and surveillance. Empirically, the paper uses interviews held with urban nightlife visitors to uncover practices of smartphone use. This qualitative and explorative study contributes to the concept of participatory surveillance by discussing to what extent smartphone-users’ actions and motivations can be seen as forms of surveillance and how that influences these actors in a (nightly) public space. We finish by setting out directions for studying mobile technologies of the self.

## Introduction

In recent years, a number of scholars have explored ways of understanding surveillance that challenge dominant perspectives, which see surveillance as organizations, power relations and inspire negative, Big Brother-type emotions. This direction of post-panopticism (Boyne 2000) includes the study of the experiences of the subjectivity engaged in surveillance practices, e.g. surveillance as resistance (Ball 2005), empowering exhibitionism (Koskela 2004) or participatory surveillance (Albrechtslund 2008). Our ambition here is to further explore how the subject participates in surveillance situations with a particular focus on how users experience everyday tracking technologies and practices. Of particular interest to us is the recently ubiquitous presence of smartphones and the emergence of wearables connected to them. The main purpose of this paper is to provide a systematic and empirically grounded analytical understanding of how individuals relate to technologies which pervade everyday life activities. The pervasive use of surveillance technologies in intimate contexts of everyday life is a critical and, at the same time, an underdeveloped issue. Systematic scholarly inquiry into this issue has the potential to significantly improve our understanding of the motives and consequences of the deep infiltration of technology into contemporary life in a networked world. Understanding these practices is a precondition for improving public policies and technology design to promote privacy protection as well as user empowerment. We focus on how the self is enacted, negotiated and maintained in an environment of increasing and elaborate practices of tracking via mobile technologies. First, we explore and analyze the theoretical implications of participatory surveillance practices in relation to smartphones. Second, we study the case of smartphone users in the nightlife district of the city centre in Rotterdam. This includes an understanding of media consumption in the public sphere. Third, we use our findings to contribute to a research agenda for surveillance studies in relation to the self.

## The Smartphone as a Platform for the Self

### Governance and Responsibility

The combination of smartphone cameras and social media platforms is relatively new. The smartphone has become a platform for both media consumption and consumption through media. Ling and Yttri (2002) and Green (2002, 2006) have shown how the smartphone changes the public sphere in terms of mobility, identity and experiences of geography, to name a few, often stating the positive effects of mobile media in public space: “The metamorphosis of space and time has consequently modified the statute of the presence and absence of individuals in social space, the relation of citizens with public space, and has interacted notably also in the role played by the mobile in the strengthening of the democratic process” (Green 2002). Although events such as the Arabic spring can be seen as providing evidence for this claim,^1^ our focus is on the smartphone in an everyday setting, specifically in Rotterdam, a large European city. In that light, the smartphone seems harmless and is indeed often interpreted as a personal tool for empowerment.^2^ However, even in contexts such as the Quantified Self-*hackatons*,^3^ smart-metering apps and sports trackers, besides all positive accounts of independence and access to knowledge in forms of self-tracking, there are multiple sides to the story of surveillance. As a contrasting guideline through this paper, it is informative to introduce two recent and strong discourses in theorizing surveillance. One is that of empowerment, in which democratization and a Do-It-Yourself (DIY) mentality are seen as force that drives self-surveillance via the recording and sharing of data and insights of technology use that is not necessarily commercially explored or government controlled. Another is of more dark nature and argues that these DIY-, self-tracking and monitoring applications (apps hereafter) are a way to lure users/citizens into a model of responsibility dependency and controllability of companies and governments alike. By subjecting ourselves to tracking and sharing of data, we engage in forms of governance where tracking potentially will transform from voluntary to obligatory, leaving increasingly less space for opting out or of self-determination.^4^

To exemplify this move towards *responsibilisation* (cf. Hinds and Grabosky 2010), in a recent campaign by the Dutch government a set of guidelines was presented on how to act in case of an emergency or incident in the public arena.^5^ Besides staying with the victim and remembering perpetrator features, taking pictures or making movies of the situation was put forward as a ‘call-to-arms’ from the government to exercise responsible citizenship (see Helgesson 2011). This example shows that, although smartphones are not introduced as surveillance devices, the possibility and potential responsibility for citizens to act with this tool in the public space has already been called upon by local governments in Dutch cities. In the campaign it is assumed that the smartphone, equipped with a camera, is something everybody carries and is able to use. Moreover, it is assumed that the act of taking pictures or making movies in public spaces is socially accepted. Such campaigns point to a new form of surveillance in public spaces, where governments are relying more and more on ‘citizen responsibility’, rather than relying on local governments and police to be responsible for safety and security in those public spaces. Taking pictures and making movies thus becomes a way for citizens to exercise their responsibility.

### Surveillance and Participation

The smartphone camera can be interpreted as a ‘potential helper’ in surveillance and monitoring. In surveillance studies, the use of mobile cameras by citizens is often considered as a form of *sousveillance* (the use of cameras by citizens to watch other citizens or as inverse surveillance specifically and purposely watching the watchers—Mann et al. 2002; Koskela 2004). However, these theories might not necessarily only be interpreted as positive or empowering (see f.i. Timan and Oudshoorn 2012). Rather than contesting or ignoring surveillance, the uploading of user-generated data such as movies or pictures that are somehow shared and made accessible to others via f.i. social media platforms can serve a complementary role in the *surveillant assemblage* (Haggerty and Ericson 2000). Theoretically, we can speak of *participatory* surveillance (Albrechtslund and Dubbeld 2005; Koskela 2011). Albrechtslund (2008) sees this participation as potentially empowering: end-user-or citizen engagement in surveillance contributes to a democratization of surveillance technologies.

Although participation in surveillance can occur in many forms, we focus on the making and sharing of footage (pictures or videos) recorded by visitors in public nightlife districts. Users of smartphone cameras in these spaces might be active in *night*-*logging;* creating footage of what happens on a specific night (see Mann et al. 2002). The sharing of such content and making it available to others implies an act of subjection to potential surveillance; citizens volunteer not only to watch but also to be watched. By whom the footage is watched, and where it ends up, is out of control of the subject who shares his or her data. Equally important, participation can happen intentionally and unintentionally; the content-maker may or may not have intended the footage to serve specific surveillance purposes. Once the record-button is pressed, one is in some form or another participating in surveillance by recording a human activity of that night out. Once material is shared, it becomes researchable and indexable by many other actors.

If the act of recording a human activity can be construed as empowering and as part of a bottom-up, counter-surveillance^6^ discourse, the act of sharing also brings about a set of new consequences. The advantage of going beyond the concept of participation as merely positive is that this phenomenon can be re-evaluated in light of critical thinking in surveillance studies (Koskela 2004; Albrechtslund and Lauritsen 2013). Treating participation in symmetrical terms allows us to shed light on the degree to which and how citizens-*cum*-smartphones as responsible and made-responsible actors are co-opted into the surveillance and production of safe public spaces. In a similar vein, this can be argued for forms of self-tracking, where the pressure to take responsibility of one’s own being is more and more being mediated. Tracking apps, for instance in sports-tracking or healthcare apps, use a variety of measures and types of data to gather and compose an image to be fed back to the user. This could entail location data, images or sound, but also more subtle, or at least less obvious data such as heart-rate, food intake, the quality of one’s sleep, or combination of different types of input data to make analysis and to provide feedback in the form of graphs or data visualisations in the interface. The point is that the smartphone and its apps are (almost) constantly carried along with the user and increasingly used in daily actions and places: they become *datafied*. Another important aspect of thinking about surveillance and subjectivation in this setup is that these smartphones are also almost always connected to another technological infrastructure with which they share data (Internet, GPRS, GPS, etc.). This is relevant because, in thinking about the user and the interface of the smartphone, this new smartphone-user *hybrid* (Latour 2005) does not only alter human-technology relations within the hybrid (meaning between the user and the device), but also expands to other places and spaces: the hybrid is embedded in a larger network. For example, when taking a picture of a night out and sharing this on Twitter or going for a run and posting that data on Facebook, not only does this change the actual activity of going out or going for a run, it also influences the networks this user-mobile is embedded in. A friend can post a comment for instance on the “great run”, or a local police station can take an interest in the image of a night out because just at that time there was an incident in the city centre and the image (potentially) holds relevant information. Leaving the legal debate on the boundaries of third-party data use^7^ aside, the point to make here is that this third-party watching does change relations between the user, the device and their connected networks.

### Subjectivation and the Mobile Self

A major trajectory in the Surveillance Studies literature (Foucault 1977; Lyon 2001) is the understanding of surveillance as a tool for identification and categorisation. Many streams of information flow together to form a “data double” (Poster 1990), a powerful tool in the hands of corporate business and government (Fuchs 2010). It is a main assumption that surveillance is an influence external to the individual, which seeks to control and discipline, entailing a risk of exploitation and privacy invasions. There are two aspects that emerge here in the context of smartphones and the (self) tracking culture. First, when individuals proactively use forms of self-tracking technologies, surveillance relates to individual experiences, motivations, and perceptions: they have become a part of processes of constructing a subject—what Foucault called *subjectivation* (Foucault 1988). Second, shifting the focus from a governmental—to an individual perspective also implies a shift in the understanding of the individual as a passive receiver of surveillance to that of an active initiator. The individual engaging in tracking differs greatly from the supervised prisoners of the Panopticon structure described by Foucault as always objects for information, but never subjects in a communication (1977, pp. 195–228). Surveillance is always an enactment of power in the sense that it is a mediating technique in practices of governance (Foucault 2007; Klauser and Albrechtslund 2014). To explain this, Foucault employs the image of the Panopticon, Jeremy Bentham’s model for prison buildings which enables the guards to watch everything without the prisoners knowing if they are being watched. Foucault describes *panopticism* as a new political anatomy where discipline replaces the earlier sovereign power (e.g. the king). In the place of the sovereign was put a more subtle and hidden authority that exercised its power by objectifying and generating knowledge from those the authority desired to control (Foucault 1977). Governance takes place in all aspects of society, involving e.g. the state’s government of its citizens, individuals’ self-governance in accordance with social norms and values, and the interplay between society as a whole and personal behavior control—providing what Foucault calls “technologies of the self” (Foucault 1988). In this line of thinking, individuals have the means to transform or improve themselves through manipulating their own bodies, thoughts, or ways of living in accordance with society’s ideals.

In that sense, we must consider the individual not only as object of surveillance but also as an acting subject of surveillance. This acting entails both the making and the sharing of data, be it personal (for example a “selfie”), or peripheral data (for example a selfie with other people in the picture). Sharing collected personal data with peers online changes the individual’s reasonable expectation of privacy (Nissenbaum 2010), and issues of ethical responsibilities regarding data ownership, commodification and sharing practices also become pertinent (Fuchs 2010). What are the ethical scenarios and risk of privacy invasion connected with individuals tracking themselves or their family members for personal purposes? Further, the increasing documentation, quantification and broadcasting of intimate details of self-change, the dynamics of performing and producing subjectivity (Papacharissi 2011). What role do potentially fragile technologies play in the mediation of the self, especially when we think of surveillance as a method of communicating or monitoring oneself and others? Although this is part of a larger research agenda, a start is made here by looking into how users of smartphones see their daily—and nightly—use in relation to surveillance.

## On Users and Smartphone Camera Practices

Taking the above theoretical points into consideration, we must ask if, how and where these instances of self-surveillance and/or participatory surveillance take place. Do citizens, as users of smartphones accept, modify or resist different modes of tracking and sharing of their smartphones and its uses, and do they explicitly relate this to ideas of *sous*-veillance, counterveillance, or see them merely as a form of entertainment? In this smartphone-user hybrid, what actions are allowed, and taken by users when it comes to making and sharing data? And if so, on what kind of a platform do they share this data and who do they think owns this data? A series of interviews with citizens of a night out in the city of Rotterdam will be analyzed, in which topics of participatory surveillance were discussed. The situatedness of this research, the night, and a city centre, are chosen because it is in these places that most contrasts and conflicts of interest can be found when thinking about citizens, public space and surveillance,^8^ as the most extreme forms of behavior in general. If citizens would make a link between surveillance and smartphone use, this would be an obvious situation. Moreover, because, of the smartphone, something else happens in light of surveillance, that can be considered new. Besides the hypothesis that the smartphone camera empowers citizens because they can ‘film back’ and ‘report’ on the spot, the recording and sharing of a night out now also stretches over space and time. Not only can the footage be shared and reviewed later via for example sharing on social network sites, it can also stretch the place of surveillance into the realm of the digital.^9^ Therefore, in the analysis, a separation is made between the smartphone camera and data use *during* a night out, and *after* a night out.

Interviewees have been recruited via snowballing (asking the interviewee to provide names for other potential interviewees). The interviews were conducted in the city centre of Rotterdam. The interviews were held in the nightlife district, and audio recordings were made of the interviews with permission of the interviewees to record the interviews and to transcribe the recordings. The interviewees ranged from 17 to 30 years old. The interviews explored whether and how smartphone cameras are being used and to what extend interviewees connected this use to topics of safety, security or surveillance. After transcriptions, TAMS analyzer software was used to explore the interviews.^10^ A topic list and a code scheme were developed by close-reading the transcriptions from the interviews. Queries were performed on the transcriptions across interviews using the topic list. For reasons of anonymity, fake names, or only first-names are used in the transcriptions.^11^ We use interview excerpts^12^ to unpack and elaborate how smartphone use changes how the self is enacted, negotiated and maintained in a nightlife setting.

### Smartphone Use in a Nightlife District

#### Frequency and Types of Use

In order to first grasp if we can actually assume a smartphone-human hybrid and consequently to establish how users use their smartphones, it is important to know if the participants bring their smartphone on a night out at all and if so, how often they use it. Christina explains:

C: Yes, well, most of the time I don’t (take my phone with me for instance). Often, when, uhm, you are with someone or you are meeting up with someone you don’t know that well, I use it to kill time. That I do use it for […]

In a similar fashion, Lucas states he takes his phone with him regularly:

L: I always carry my phone. And of course, it would be that, might you get lost somewhere, that you can make a phone call. That’s quite useful.

Another participant, Marieke, states:

M: I always take my money and my phone with me. I think I use it about four times a night.

In fact, all of these participants mentioned the smartphone as a part of a standard checklist of things to bring before closing the door (the other two are keys and money or a bankcard). The phone is used for many things sometimes to look something up, sometimes to kill time. The smartphone here is not only a multi-functional tool, it also serves as a toy or as distraction when being in a bar. This shows that non-deliberate or non-planned use also occurs. Concerning planned, or conscious use, in asking how often the smartphone is used during a night. Bart explains:

B: Well, I use my phone to call obviously and to text where everybody is, sometimes to take a picture, and… sometimes to check or exchange things online such as someone’s phone number, to check mail. I do take my phone with me into the club or bar, where, if we sit down, it is often at the table because I like to be able to see if I have a new message, for instance. If I have to go to a toilet I take it with me.

Lucas shares a similar observation:

L: If you’ve got nothing to talk about with someone, or just for the time being. It has become sort of a replacement for having a beer, or something. I mean, very often, it’s just nice to hold on to something.

Both mention a variety of functionalities of the device that they use. These use practices exemplify how the smartphone not only invites users to follow the program of actions as described in the phone, but that it supports non-‘functional’ actions as well (checking, holding). In this respect, the smartphone must be considered as a ‘multiple’ artefact, or platform, rather than being single-purpose and clear cut. If indeed the smartphone in the night is used in multiple ways, one of these ways is to take pictures or to make movies.

#### Camera Use

Questions of camera use become important in light of surveillance in two ways; one is the taking of pictures or making of movies during a night out and the second is the reviewing and sharing of this footage. The question is how and why these nighttime visitors make and share footage. Lucas states:

L: Well I do make pictures or movies during in-between moments.

Christina, however, resists the use of the phone camera during a night out:

Chr: I am not the kind of person who grabs her phone and turns on the camera when something happens.

For her, the activity of going out and the possible use of cameras by visitors are not coupled tightly. Where for Lucas it is. She refers to ‘when something happens’, which she positions in the interview as something negative, and not fit or appropriate for recording. To questions about smartphone camera use and sharing, Bart responds:

B: Uhm, well, quite regularly… I’m not the one who records everything and puts it on Facebook.

He links using a camera directly to sharing of content—an activity from which he distances himself. As to why he takes pictures, Bart states:

B: I usually take pictures of unexpected events, that I really like to do. If, for instance, there is something in a bar or in on the street that reminds me of someone, then I think: “I have to take a picture!” […]

Wouter has a similar response to why one would take pictures in public space, especially during a night out:

W: I don’t do that in a group-context. If I make pictures, it’s often detail-things; pictures of things that struck me, or that I found funny. Or if it reminds me of something or, if I see a name or an object that reminds me of someone, then, I make a picture of that.

Both Wouter and Bart rework the action of taking pictures into capturing ‘unexpected’ events and atmospheres. They refer mainly to taking pictures and both do not mention making movies at any point. So far, no explicit links have been made to surveillance or incidents and personal cameras. In that sense, the idea of consciously participating by recording incidents or fights does not seem to be a part of the use practice of smartphone cameras for these users.

#### Responses to the Act of Taking Pictures and Filming

In order to explore the making of footage from another perspective, the interviewees were asked whether they have had experiences with responses of others to their use of their smartphone camera? And vice versa, did it trigger certain responses in them to be filmed, and it that sense, watched by other citizens via smartphone cameras? Suzanne explains:

S: Well, yes, if someone would really take a picture of me, then I would feel uncomfortable. Unless they would have asked permission before. Of course it happens that you end up in someone else’s picture accidentally. This also happens when you take a picture yourself and then you also do not go around asking everyone in the street for permission. So in these cases I do not think that others have to ask me for permission.

Here, a camera etiquette is introduced, in which boundaries, also hinting at the tension of what happens and what is allowed legally to happen. If we consider camera usage also part of self-tracking, or self-recording, where can boundaries be drawn on making others part of that recording? It can be expected that the use of cameras and the sharing of content varies widely amongst users. For instance, the awareness of a camera in her close proximity is a reason for Christina to be a non-user:

Chr: […] I have the impression that taking pictures is an interruption […] I’d rather be experiencing a situation than to capture it […] (the camera) is in between the situation and me.

For Marieke responses on camera use tended to be positive:

T: Have you ever had responses from people?

M: Yes, often positive responses.

For Marieke the response of the person(s) in front of the camera is something normal: as something to not be worried about, whereas Christina points at a distortion of the moment. This points at a larger question of not only why smartphone cameras are used, but also what this does with a situation. Bart defines a very specific situation:

B: If I indeed get a response, which is rare, it is because people don’t like me taking pictures of something […] nobody ever comments on me taking pictures in bars or public spaces. Also because, I think, I don’t take pictures of (groups of) people. Although I think it is allowed to do so, I focus on pictures in which I can recognise myself, so pictures of me and my friends.

Here another perspective is taken on the norms of smartphone camera use. Bart shows a level of anticipation of the consequences of camera use in public nightlife, not taking the making of movies or taking of pictures for granted at all. Nonetheless, he also remarks that it is not really necessary to take the ‘subjects’ of camera use into account in public spaces or bars, as nobody really seems to care in those specific contexts. The reasons for using a camera, mostly to take pictures rather than to shoot videos, also vary between these users, however, Marieke and Bart both provide the reason of capturing context—to memorize the night. In the above examples we see how actual behavior in public space alters due to different expectations and manifestations of smartphone use.

#### Types of Content and Use of Footage in the Night

Besides memorizing the night (which in some cases resonates with surveillance because it concerns collecting visual evidence of human activity)—to recollect what happened, there might be other reasons to share pictures. One interviewee points out that the focus of his footage is on things rather than people. Therefore when he takes pictures, it not really relevant for those surrounding him:

W: So, when I take pictures, it’s often of things and surroundings. So it’s not that I attract attention of others’ by taking pictures.

Besides taking pictures or making movies, the program of action of the mobile camera allows for direct reviewing as well. Bart was the one of the few who mentioned reviewing footage during a night out. The main reason for directly reviewing images is linked to fun and entertainment:

B: This is highly dependent of the scene I am in. Musicians I know, for instance, care a lot about exposure, so they like to review pictures directly. In my other social group, it is more a thing to review after.

In this case, users mainly capture, store and carry information that might be valuable later on. Also here, none of the responses mentioned reviewing in light of incidents or in any surveillance-related manner. Direct sharing or posting of footage on the Web, or on any other sharing platform, did not occur either. Reviewing images was done to ‘check the picture’ on the spot, or to retrieve information (e.g. a map or bus schedule). Seeing how immediately sharing footage is not something the studied participants did, the next is to explore how and where footage might otherwise be shared.

### Role of Footage After the Night: Sharing Nightlife Footage

#### Reasons and Places of Review

When urban nightlife consumers, including the study participants, take pictures or make a video on a night out, they presumably have some goal or intention in mind for this content. However, in the act of sharing, content and intention become separated. Where any third party gains access, control over this data is lost. In this section, it is questioned to what extent sharing of content takes place, and to what extent the interviewees connect this to surveillance or surveillance-related topics. Marieke has never faced any negative consequences from sharing content or watching others’ content:

M: I like it (reviewing other’s pictures).

T: You have never heard negative stories?

M: Well, not really. I like to see pictures of others; it gives me an idea of the night.

For her, pictures serve the purpose of recalling a night. Not all pictures, however, end up being deemed relevant, important or nice to share and relive the night. The selection and sharing process is a group process in her friend group:

M: Yes, well, the ones we like end up on Facebook or Hyves.

T: And who decides this?

M: We do that ourselves.

T: […] And if you review images, do you do that alone or with friends?

M: Well, it depends. If we see each other at school the next day, we review collectively.

Other camera users, such as Lucas, points out some moments of reviewing images the next day, however, the context is individual:

L: It’s more like, how to phrase it… often you forget that you’ve made pictures. At least, I do. And then it’s nice to review images the day after; that can be fun. Then stories reappear.

Other responses show that also here practices of use, in this case reviewing images, vary widely in term of time and moments to review:

W: Well, yes, I am not such a fan of reviewing images. Only after 5 years or something. Then it becomes interesting to me again, then it becomes fun, but close to the moment itself. It (reviewing images) is not really on my mind.

Forms of self-censorship, sharing and a set of values on what is a ‘good’ representation of that night are part of the process of participation here (see e.g. Miller and Edwards 2007). The ‘life’ of the images once they have been posted online is not a lengthy one (see Van House and Davis 2005, who mention a ‘short’ life of images for the moment and a ‘long’ life for memories). The human-camera hybrid expands far over the time of content-making. In light of participatory surveillance, questions of accessibility start playing a role, where some content made that night is reachable for others, or third parties interested in this data, and some data remains hidden. In the case of sharing via social network sites or other online platforms, processes of self-censorship and selection also play a role in determining what ends up on the Web.

#### Practices of Sharing and Deleting

Once online, footage can become accessible for others to see. However, on many social network sites, privacy systems and security measures are in place to control the scope of visibility of one’s data. If indeed user-generated content is used by surveilling parties, questions of ownership and control over one’s data arise. Actions such as tagging, deleting, or resetting privacy settings of data (in this case images or movies) are often part of the possibilities of social network sites. Interviewees were asked if they were ever asked or if they themselves asked others to delete footage:

T: Did you ever receive a request from friends to delete a picture?

B: No, never.

T: And the other way around? Have you ever asked someone to remove something from Facebook, for instance?

B: Well, I am not on Facebook, but I would check it regularly… I can recall asking someone to not publish a picture, […] but that was IP-related.

B, thus, keeps in mind potential IP (Intellectual Property) infringement or misuse of his pictures, and does not worry too much about these matters as he has deliberately chosen not to be on Facebook. Also Lucas recalls one instance of a request to delete a shared recording:

L: I uploaded a movie once and then I received a text message asking me to remove it.

Other participants, such as Wouter, showed no concerns surrounding aftercare of shared data:

W: I am not really ashamed of anything, so… In that sense, there are always people who do not want for certain things to end up on Facebook. Well, that does not hold for me.

This non-concern about privacy and the sharing of data is countered by another extreme of the spectrum of use of social media; non-use. Although Christina is aware of searching and looking up content made by friends, she has no experience with participation-by-contribution in the pool of data:

Chr: Oh, I am really bad at that. I would not even know how to upload anything.

T: And in your friend-group, is the Internet ever used to search for movies or pictures?

Chr: I think so. but I don’t do that so much. Via Facebook, maybe…

This quote highlights another aspect of participatory surveillance; social network sites allow one to be watched, also as a non-user. In the public nightlife, there is still a possibility to refuse, or react on another citizen who is pointing a camera at you or when you happen to be in the background of other’s images; once data is shared, it becomes impossible to see who is watching. Besides these online social media sites, smartphones allow for other forms of sharing. Bart sometimes shares images by showing them on his smartphone:

B: […] Yes, if I am with friends occasionally, or at work after the weekend, then I share… I show my pictures […] Just on my phone; if they have accidentally been put on my computer, then I show them on my computer […] No, no I don’t put things on the Internet […] the tricky thing is that once you upload something, you lose control over it. Especially when it concerns personal things, like pictures or mutterings […] I like to have control over my data.

He chooses to make clear the boundaries of who is allowed to be part of his visual log. An issue that emerges here is control over one’s data, with the fear that once shared, one loses control over it. Delving into this boundary of where the openness of data does become problematic, Lucas highlights:

L: It does not really matter so much to me. But at some point it does, when it concerns a work-context, in which people deem it important to keep things private. And I put more things online than most people.

Concerning sharing practices, Marieke only reflects on the purpose of images *for herself* and does not mind using social media sites, whereas Bart values *control* over data and consciously keeps his footage local, thereby also selecting and determining who gets to see his content. Marieke and Wouter contributed to the pool of potential surveillance data and do not care about the after-life of their footage. Lucas points out that in cases of a work context, privacy and control over data becomes an issue. Also non-use due to a lack of understanding the mechanisms of social media occurs, as exemplified by Christina.

Sharing is thus multi-faceted and does not necessarily mean opening up to any third-party; the hybrid of visitor-camera can take on many forms, also in notions of sharing. The smartphone camera allows for multiple ways of dealing with footage after a night. The temporality of the purpose of footage is a theme that emerges in relation to participatory surveillance and mobile devices. Not having discussed social media sites in relation to privacy settings and control over data in detail here,^13^ the interviews did provide data that allows for discussing the boundaries of use of data.

#### Footage Ownership and Boundaries of Use

When Christina was confronted with a case of police surveillance (a scenario in which the police would use YouTube footage uploaded by urban nightlife visitors such as herself to reconstruct an accident and identify suspects), she pointed out the following:

C: Well, that’s a human response I think, to combine things and that you try to achieve the best possible results with the means available. If you look at it from that perspective, it works- it is really a one-way stream. But the other way around does not work; that as a public you would have the rights to access police footage – if you ask me.[…]. If the police would use YouTube to fix a problem or find a perpetrator, that’s fine. But the idea that information is so open and exposed, thats a bit, uhm, double.

T: And who should protect that limit, on what is open and what not?

C: You can’t! That’s the whole infinity of the Internet, of media, for me. And I would not consider myself as a person who can protect that border.

Her view on participatory surveillance is double-sided: it consists of both the idea that it is hard to be against the use of third-party photos and videos for solving crimes, and the view that it is not desirable for all information to be open and accessible. She continues by explaining that in her view footage made of public space can belong to the police, but footage made by the police can never be accessible to the public. This is indicative of the role of the new hybrid of visitor-camera: the power relation to official, organizational surveillance^14^ remains asymmetrical. Marieke has her own view on (non) participation:

M: What I put on my smartphone does not end up on the Internet unless I want to.

T: So you control the footage?

M: Yes.

T: What if the government would make use of your footage?

M: Well, I can make a picture of an incident. But they don’t own nor control these images.

M: […] If it serves the right purpose, or goal, then it’s no problem, I guess.

Although sharing pictures of the night is part of her ritual of going out, she still feels in control of her data, and clearly she is the one who decides what goes online and what does not. More importantly, the action of sharing does not entail that the footage is no longer her property. However, when it serves a good purpose, she is fine with police looking at her images. This point is not shared by all participants. Wouter points towards the possibility to de-share:

W: So, the moment you become recognizable, you should have the possibility to remove it, that’s how I see it. The moment you become recognizable via technological means, you should also be able to delete that- to say that you do not agree.

In this quote, the boundaries of use of other’s data for surveillance purposes is set to a reciprocity of possibilities, or options to deal with data; of being able to negotiate over one’s own data. The smartphone does not alert users in any form about potential consequences of sharing their data. Not informing the user about the possibility to de-share, or to disallow the use of data for third parties leaves the data of smartphone users relatively unprotected. Neither the mobile devices nor the platforms on which content is shared, provide the user with information regarding image ownership or the consequences of sharing. Possibilities for sharing content via these mobile media devices are abundant, which is in sharp contrast to the total lack of guidance on how to control ones’ image ownership or how to delete or de-share. The diversity in use practices shows that smartphone user and sharing content is interpreted in a high variety of ways; this exemplifies the complexity and the multiplicity of ways in which participation, subjectification and instances of surveillance occur in public space.

## Conclusions: Directions for Studying Mobile Technologies of the Self

Contributing to existing research on the experience and influence of smartphone technology on public spaces (see f.i. Castells et al. 2007; Green 2002; Ling and Yttri 2002), this is another small step to expand the scope of this research by not only looking into positive effects, but to also incorporate new threats for privacy and safety via forms of surveillance through smartphones and social media. Although making or sharing of footage occurred amongst the participants in this study, it was not in any way linked to recording incidents or disorderly conduct. However, the smartphone camera is being used and footage is shared. This shows that participation in the form of making and sharing audiovisual material of the night does in fact take place. In that respect, the smartphone-user hybrid becomes a touch point for surveillance in the (nightly) city. This is in line with the realisation in surveillance studies that we need to look at new places of surveillance; ones that go beyond the classical structure of CCTV and citizens as subjects of surveillance.

Looking into sharing practices can shed light on how, where and when processes of subjectivation take place. Surveillance via smartphone cameras does take place, where we saw instances of self-correction (only talking images of things, or deliberately not focusing on faces) and deliberations before actually using the smartphone camera (would I like to be treated in a similar fashion). Surveillance and subjectivation also turned up as an issue in cases of sharing data made during a night out: negotiations with peers accompanied by the fear of losing control over one’s data turned out to be key points in practices of creating and sharing footage. In that sense, contrastingly to the Quantified Self and other data-sharing movements, where sharing is seen as an empowering activity,^15^ social media can also be interpreted as disempowering the individual (see f.i. Mostmans et al. 2014), this self-reported and shared data of a nice night out might have unintended consequences, resulting in—and this reflects partially in the respondents’ answers—constant deliberation on if, how and where to share what with whom. Self-tracking here might not take place via numbers and repetitive check-ins, smartphone use in the nightlife context might lead to other forms of ‘self-tracking’ and representation online that does adhere to the QS-adage of more data is more knowledge, however, this is not necessarily in favour of the individual sharing that data. Even when the meaning of recording of a public space is single (e.g. to monitor, or ‘for fun’), in the moment of sharing, meaning and purpose can get lost, or multiply: what might be fun for an individual citizen to share, might be crucial surveillance information for a local police station, or for 3rd party commercial entities such as advertising companies who see value in this data. A central issue at stake here is that of power and surveillance in the intersecting networks of stakeholders and users. When talking about governing or regulating this type of surveillance, where to place individual yet networked mobile devices that contribute to surveillance by sharing all sorts of data such as images, movies, location? The respondents did not feel a part of a surveillance network, yet as soon as images are made and shared, updates on incidents are tweeted or status—updates given, they are in that network already. New questions of governance, responsibility and accountability arise here, where a challenge for researchers of surveillance lie in investigating these new networks^16^ of users, operating systems, software engineers, third-party data snoopers and local police-officers who scan social media, to name a few.

Another aspect is that of subjectivation and the self. Despite the act of taking pictures and sharing them on social media is made deliberately and consciously, it does change how the individual citizens acts; where in our examples, the subject of the camera hardly rejected, we did see instances of both watcher and watched adopting different strategies to deal with smartphone cameras in public spaces. The examples showed that it is mostly about taking pictures of oneself in a specific social context, or to record certain things of interest, or just a reminder and an external memory of a night out. These can be seen as different forms self-improvement. As mobile technologies move from these conscious functionalities and actions of self-improvement toward tracking and monitoring apps, the conscious roles of both watcher and sometimes watched also change. These apps often entail repetitive, mundane practices or techniques of recording and tracking, which shape how users perceive themselves and their surroundings. As such, tracking technologies function as tools for governing the self (Foucault 1988). The smartphone as a self-tracking device then becomes a more autonomous agent in sharing that self by constantly recording and sending out representations of that self. It adds to the literature on surveillance and self-tracking a qualitative account of what happens in the night concerning smartphone usage. This paper tried to show that data is actually being made and shared, but that this making and sharing is not seen by the participants as forms of surveillance but rather as forms of self-tracking. However, this self-tracking and sharing of data can lead to third-party watchers, be they government, or corporate surveillance. Users should be more protected- and more wary—when sharing data.

## Footnotes

1 See f.i. Chen, *How networks changed the world* (2011). Network, IEEE, or Mihailidis (2011) New civic voices & the emerging media literacy landscape—The Journal of Media Literacy Education as examples of a myriad of often techno optimistic views on the role of social media, smartphones in the Arabic Spring movement M.

2 Cf. Hofheinz (2005).

3 A hacking marathon, formerly organized by hackers in an attempt to democratise technology by making it accessible for ‘the public’, nowadays often hackatons are events sponsored by companies to get ideas for free. See f.i. http://www.hackathon.io/events.

4 A recent overview-piece on the end of our privacy under the flag of empowerment can be found in Science magazine: 30 mag, January 2015, Vol 347, Issue 6221.

5
https://www.nederlandveilig.nl/pakdeovervaller/.

6 Meaning to surveil the surveillors by ‘monitoring back’ or ‘returning the gaze’. See also Mann et al. (2002).

7 Although this is a highly relevant topic, since we see more and more the collapse of surveillance technology and instruments with those of consumer electronics and with that a blurring of boundaries and roles of surveillance, it goes beyond the scope of this particular paper.

8 See van Liempt et al. (2012).

9 An example here could be police finding images or filmed footage on a digital repository after the fact.

10 See TAMS analyser website: http://tamsys.sourceforge.net/.

11 These transcripts are available upon request. The T in the transcripts represent the interviewer.

12 See f.i. Hine (2007).

13 Although highly relevant for expanding the type of research as presented in this paper, this is beyond the scope of this paper.

14 See Tække (2011).

15 Especially in the Quantified Self movement, the sharing of data is seen as crucial for the individual and the collective of QS: by sharing data, algorithms, thus potential solutions become better and more robust and this is turn helps the individual.

16 A very good start in visualising these networks has been made by the DMI group at the UvA, via their Tracker–tracker-tool, which allows you to see who else is looking at your social media data: https://wiki.digitalmethods.net/Dmi/ToolTrackerTracker.
